# Artificial Intelligence in Peripheral Artery Disease Education: A Battle Between ChatGPT and Google Gemini

**DOI:** 10.7759/cureus.85174

**Published:** 2025-06-01

**Authors:** Shriya Patel, Julie Ponn, Thomas J Lee, Arthi Palani, Heather Wang, Julius M Gardin

**Affiliations:** 1 Department of Internal Medicine, Rutgers University New Jersey Medical School, Newark, USA; 2 Department of Internal Medicine, Division of Cardiology, Rutgers University New Jersey Medical School, Newark, USA

**Keywords:** artificial intelligence in medicine, chatgpt, google gemini, online medical education, peripheral artery disease (pad)

## Abstract

Background

Peripheral artery disease (PAD) is a prevalent yet often overlooked manifestation of atherosclerosis that significantly contributes to cardiovascular morbidity and mortality. With the increasing reliance on artificial intelligence (AI) for medical information, it is essential to assess the accuracy and readability of AI-generated health content, especially with regard to common cardiovascular diseases.

Objective

This study evaluates the accuracy, completeness, and readability of responses generated by OpenAI’s ChatGPT (San Francisco, CA) and Google’s Gemini (Mountain View, CA) when answering common questions about PAD. AI responses were compared to Cleveland Clinic’s frequently asked questions (FAQs) on PAD to assess the reliability of AI-generated responses as a patient education tool.

Methods

ChatGPT 4.0 and Gemini 1.0 were prompted in three formats (no prompt (Form 1), patient-level prompt (Form 2), and physician-level prompt (Form 3)) before answering 19 questions from Cleveland Clinic’s FAQs on PAD. Responses were categorized as correct, partially correct, or incorrect based on percent content alignment. Readability was assessed using the Flesch-Kincaid (FK) grade level, and word count differences were analyzed. Chi-square tests and one-way analysis of variance (ANOVA) were used for statistical analysis, with a significance threshold of p < 0.05.

Results

ChatGPT provided 70% correct and 30% partially correct responses, with no incorrect answers. Gemini provided 52% correct, 45% partially correct, and 3% incorrect responses. ChatGPT performed significantly better in accuracy, with a p-value < 0.05. FK analysis showed no significant readability differences between the two chatbots (mean FK grade: ChatGPT, 10.81; Gemini, 10.73), although both were higher than the recommended reading level for patient education. ChatGPT’s responses were significantly longer than Gemini’s, with a p-value < 0.0001.

Conclusion

Both ChatGPT and Gemini provided mostly accurate and comprehensive responses to commonly asked questions about PAD, demonstrating their potential use as supplementary education tools for patients with appropriate provider oversight. However, the grade reading level of these materials exceeded the recommended reading levels set forth by national guidelines, which warrants improvement in AI-driven health communication. Given the growing reliance on AI in healthcare, further research should explore ways to enhance AI-generated medical content for broader patient accessibility and evaluate its impact on patient outcomes.

## Introduction

Peripheral artery disease (PAD) is a manifestation of atherosclerosis and is a major contributor to cardiovascular morbidity and mortality [1]. PAD affects over 230 million people worldwide, with an estimated prevalence in 2015 of 5.6% worldwide [1,2], with 10 million adults over age 40 affected in the United States [3]. Risk factors for PAD include traditional cardiovascular risk factors, e.g., smoking, age, and diabetes. Unfortunately, PAD has traditionally been overlooked compared to coronary artery disease and stroke, potentially owing to its nonspecific symptoms and lack of awareness by patients, warranting a stronger emphasis on education and accessible information given to its potential for significant morbidity [1].

Online educational information has become widely used by patients to retrieve information on their medical conditions. A Health Information National Trends Survey (HINTS) conducted by the National Cancer Institute in 2022 showed that 84.1% of the US adult population used the internet for health or medical information [4]. With the advent of artificial intelligence (AI), chatbots, and the accessibility of online information, this number is only expected to rise in the coming years.

In this study, we assessed the accuracy, completeness, and readability of AI-generated responses from OpenAI’s ChatGPT (San Francisco, CA) and Google’s Gemini (Mountain View, CA) compared to Cleveland Clinic’s frequently asked questions (FAQs) by patients about PAD. Cleveland Clinic is a well-known source that provides reliable, clinically accurate, and evidence-based content for patient education. By analyzing AI responses compared to this Cleveland Clinic standard, we sought to evaluate whether AI provides accurate, comprehensive, and readable information to patients about PAD.

## Materials and methods

OpenAI’s ChatGPT and Google’s Gemini chatbots were each prompted three separate times with a set of 19 questions from the Cleveland Clinic’s FAQs about PAD (Table 1). ChatGPT version 4.0 and Gemini version 1.0 were used for all responses. All questions were asked on January 21 or 22, 2025. Chatbot prompting was randomized, and each query was run once.

**Table 1:** Questionnaire from Cleveland Clinic’s FAQs on PAD Credits: Shriya Patel, DO, Julie Ponn, DO, Thomas J. Lee, MD, Arthi Palani, MD, Heather Wang, MD, Julius M. Gardin, MD PAD: peripheral artery disease

Question
What is peripheral artery disease?
How common is peripheral artery disease?
How does peripheral artery disease affect my body?
What are the stages of peripheral artery disease?
What is considered the first symptom of peripheral artery disease?
What are the typical symptoms of peripheral artery disease?
What are the complications of peripheral artery disease?
What is the most common cause of peripheral artery disease?
What are the risk factors for peripheral artery disease?
How is peripheral artery disease diagnosed?
Can peripheral artery disease be reversed?
How is peripheral artery disease treated?
How long does it take to recover from treatment for peripheral artery disease?
How can I reduce my risk for peripheral artery disease?
What can I expect if I have peripheral artery disease?
How do I take care of myself?
When should I see my healthcare provider?
When should I go to the ER?
What questions should I ask my doctor?

Prompts were as follows: no prompt (query without user context) (Form 1), patient-level prompt (Form 2), and physician-level prompt (Form 3). The prompts used are shown in Table 2. Responses were evaluated by one independent reviewer and scored as incorrect, partially correct, and correct based on keywords and concepts. “Incorrect” was assigned if the response included any incorrect information or if responses included less than 50% of the information included in the Cleveland Clinic’s answers. “Partially correct” responses included those that had no incorrect information and included 50%-99% of the information in the Cleveland Clinic’s responses. “Correct” responses included all information from the Cleveland Clinic’s responses, with any extra information being correct. Proportions of responses with differing scores were compared using chi-square analysis. Statistical tests were performed with an alpha set at 0.05.

**Table 2:** ChatGPT and Gemini prompts Prompts provided to ChatGPT or Gemini before asking questions

Form number	Form name	ChatGPT/Gemini prompt provided
1	No prompting	No prompting.
2	Patient-friendly prompting	I am a patient attempting to learn more about peripheral artery disease. I am going to ask you 19 questions pertaining to this. Please use language that would be appropriate for my understanding, but do not compromise on the accuracy of your responses. Be as specific as possible in your answers.
3	Physician-level prompting	I am a board-certified physician attempting to learn the most up-to-date information on peripheral artery disease. I am going to ask you 19 questions pertaining to this. Please use language that would be appropriate for my expert-level understanding of medical concepts. Be as specific as possible in your answers

For each response, the number of words, sentences, and syllables were collected to compute a Flesh-Kincaid (FK) grade level. This metric estimates the US educational grade level required to understand the response, with higher grade levels indicating more complex language usage, and is defined as follows:

$\left( 0.39 \right)\frac{words}{sentences}\text{+}\left( 11.8 \right)\frac{syllables}{words}\text{-}\text{15.59}$

FK values vary from 0 to 20, with the numerical value corresponding to the reading grade level (e.g., 10 would equal grade level 10). Significance between forms was calculated using a one-way analysis of variance (ANOVA) with an alpha of 0.05. Additionally, response length was recorded, and significance was analyzed with a one-way ANOVA and an alpha set at 0.05. An unpaired t-test was used to calculate the significance between the values provided by ChatGPT and Gemini. Statistics were run using Prism 10.0.2 (GraphPad Software, San Diego, CA). Standard deviations (SD) and confidence intervals (CI) were also presented.

## Results

Across all forms, scoring frequencies for ChatGPT were as follows: 40 (70%) correct, 17 (30%) partially correct, and 0 (zero) incorrect. Scoring frequencies for Gemini were as follows: 30 (52%) correct, 26 (45%) partially correct, and one (3%) incorrect. Chi-square analysis revealed a significant difference in the correct responses provided by ChatGPT compared to Gemini (p < 0.05).

**Figure 1:**
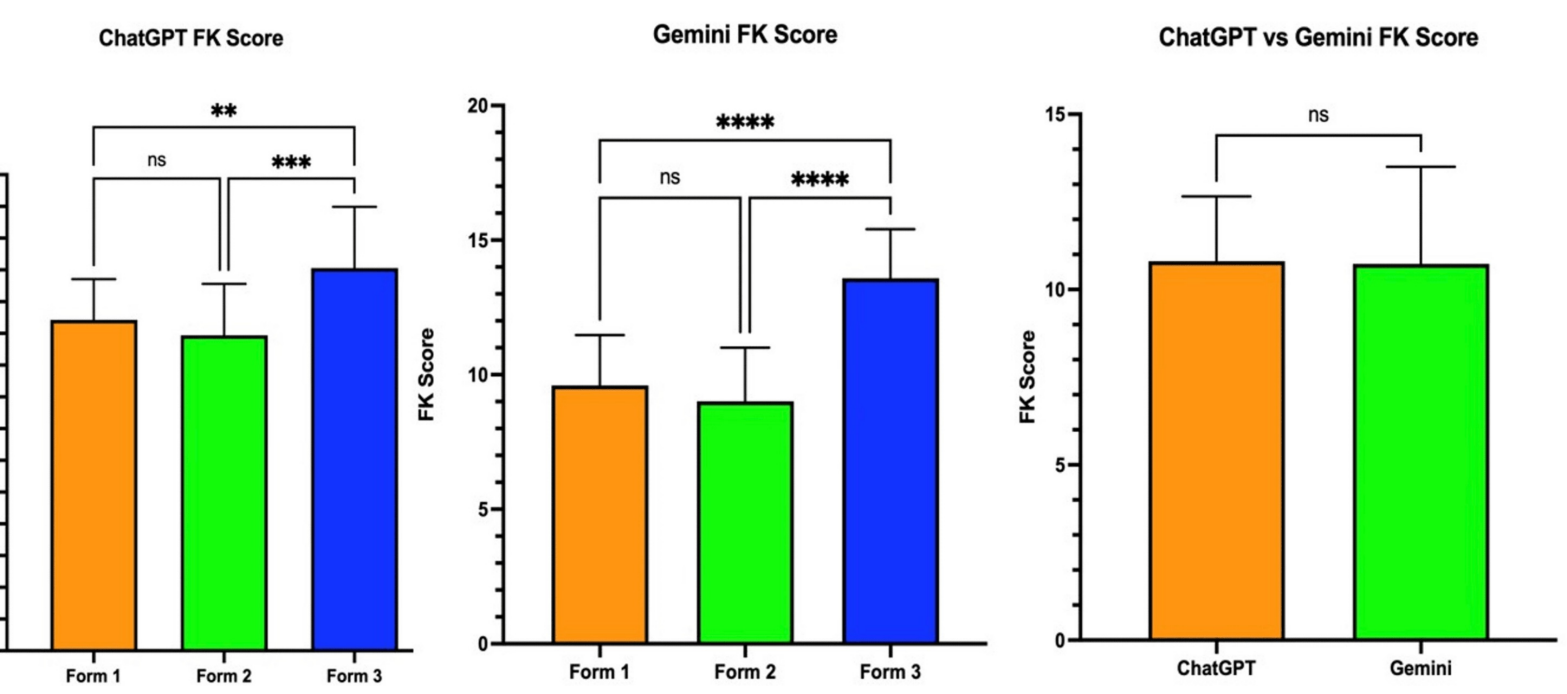
FK grade reading level comparison between all forms for ChatGPT and Gemini FK: Flesch-Kincaid, ns: no significance **p < 0.01 ***p < 0.001 ****p < 0.0001

FK reading level scores for ChatGPT and Gemini are shown in Figure 1. ChatGPT’s mean FK grade reading level was as follows: Form 1 at 10.43 (SD = 1.29, 95% CI = 9.81-11.05), Form 2 at 9.94 (SD = 1.62, 95% CI = 9.16-10.72), and Form 3 at 12.06 (SD = 1.93, 95% CI = 11.13-12.99). Tukey’s multiple comparisons test was used for comparison of the means between each form. In ChatGPT’s responses, a significant difference was found between Form 1 and Form 3 (p < 0.009) and between Form 2 and Form 3 (p < 0.0006). Gemini’s mean FK grade reading level was as follows: Form 1 at 9.6 (SD = 1.87, 95% CI = 8.7-10.5), Form 2 at 9.01 (SD = 1.99, 95% CI = 8.05-9.97), and Form 3 at 13.59 (SD = 1.82, 95% CI = 12.71-14.46). In Gemini’s responses, Tukey’s multiple comparisons test showed a significant difference between Form 1 and Form 3 (p < 0.0001) and between Form 2 and Form 3 (p < 0.0001). Overall, there was no significant difference in reading level between ChatGPT and Gemini.

**Figure 2:**
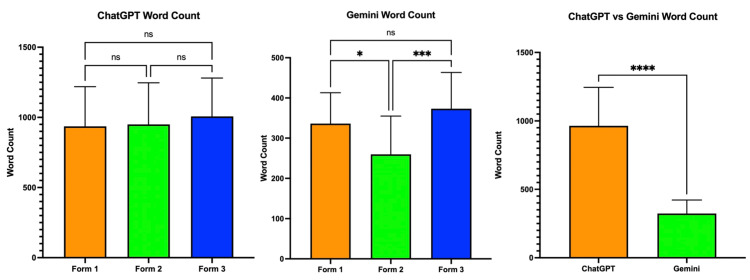
Word count comparison between all forms for ChatGPT and Gemini ns: no significance *p < 0.05 ***p < 0.001 ****p < 0.0001

Word count for ChatGPT and Gemini is shown in Figure 2. ChatGPT’s mean word count was as follows: Form 1 at 936.3 (SD = 282.5, 95% CI = 800.1-1072), Form 2 at 949.8 (SD = 296.7, 95% CI = 806.8-1093), and Form 3 at 1007 (SD = 273.2, 95% CI = 875.4-1139). Tukey’s multiple comparisons test showed no significant difference between all forms for ChatGPT’s responses. Gemini’s mean word count was as follows: Form 1 at 336.3 (SD = 76.74, 95% CI = 299.3-373.3), Form 2 at 259.7 (SD = 95.39, 95% CI = 213.7-305.7), and Form 3 at 373.3 (SD = 89.98, 95% CI = 329.9-416.6). Tukey’s multiple comparisons test showed a significant difference between Form 1 and Form 2 (p < 0.03) and between Form 2 and Form 3 (p < 0.0006). Overall, there was a significant difference in mean word count between ChatGPT (964.4 ± 280.9) and Gemini (323.1 ± 98.48) (p < 0.0001).

## Discussion

ChatGPT and Google Gemini are both popular chatbots whose uses have expanded into the domain of medicine and healthcare. Both have been utilized to answer questions about various medical topics, but few studies have assessed and compared the accuracy and adaptability of both [5,6]. While there are several studies in the literature that have compared the performance between both chatbots on various cardiology topics, this is one of the few to do so with PAD [7]. A recent study that assessed chatbot performance with PAD found that ChatGPT responses were more accurate and satisfactory in answering scientific questions and FAQs compared with Gemini [7].

While both ChatGPT and Gemini provided accurate answers, with at least 50% of responses categorized as correct and containing all information compared to the corresponding answer from the Cleveland Clinic’s FAQs, ChatGPT provided more accurate responses. ChatGPT generated no incorrect answers, while Gemini generated one answer that was marked as incorrect or incomplete, where the information provided was less than 50% of the Cleveland Clinic’s answer. Our result was similar to several other published studies that examined AI chatbot responses, with reported ranges between 1% and 5% of incorrect responses [5,8-11]. Gemini also provided more partially correct answers than ChatGPT, while ChatGPT exhibited a significant trend to provide more correct responses than Gemini, which is similar to a prior study that assessed PAD responses [7].

Regarding FK scores, there was no significant difference in all forms between either chatbot, and both had similar mean grade levels (ChatGPT: 10.81, Gemini: 10.73). There was a significant difference between Form 1 and Form 3 and between Form 2 and Form 3 for both ChatGPT and Gemini. Form 3’s mean reading level was also the highest among all forms (ChatGPT: 12.06, Gemini: 13.59), supporting the fact that physician-prompted answers were more complex and used a higher grade level. This demonstrates the adaptability of chatbots in targeting and personalizing responses to the proper audience and providing tailored answers. However, it does not follow the American Medical Association’s recommended reading level of grade 6 or below for online educational materials, grade 3-5 reading level for patients with low health literacy, or the National Institute of Health’s recommended reading level of grade 8 [12-14]. This finding is consistent with other studies in the literature that assessed the reading level for online patient educational materials for PAD [15,16]. Other credible online resources for patient education, such as the American College of Cardiology (ACC) and American Heart Association (AHA), are also written above the recommended reading level set by the NIH [17]. Online material on non-academic websites on cardiovascular topics also contained information written above the recommended grade level [18,19]. While this highlights the disparity in available health information, it shows that chatbots are on par with popular online resources currently used by patients.

Overall, ChatGPT had a significantly higher mean word count compared to Gemini throughout all forms (p < 0.0001). Within ChatGPT, there was no significant difference between the forms. Within Gemini, there was a difference in word count between Form 1 and Form 2 (p < 0.02) and between Form 2 and Form 3 (p < 0.0006). This trend was different than what was observed in other studies that compared performance between both chatbots on various topics, suggesting that the performance of either chatbot may vary based on the topic prompted [5,20]. Additionally, ChatGPT provided more correct answers than Gemini, suggesting that the increased word count may be associated with a higher educational quality.

One limitation of this study is that chatbot answers were compared to only one credible source of information and evaluated by one non-blinded independent reviewer. Future research should also compare chatbot responses with multiple credible online sources and responses from experts in the field to assess both their accuracy and credibility. Future studies may also include a larger sample size, as only 19 questions from the Cleveland Clinic’s FAQs were used in this study, as well as include multiple blinded reviewers. Additionally, questions may be asked in a multitude of ways and combinations, which may lead to different responses that were not assessed in this study. Given the amount of online information written above the recommended grade level, future studies can assess whether patient information written at or below the recommended grade level leads to improved cardiovascular outcomes. Another limitation is that chatbot performance can vary over time due to model updates, and this study was conducted only at a single time point. Strengths include an objective comparison between AI-generated answers against a reputable source that is widely available, as well as a separate analysis on the readability and complexity of the text provided. While this study demonstrated overall positive results, it is important to remember the ethical concerns regarding the use of AI tools in patient education, including overreliance on AI and the risk of misinformation.

## Conclusions

This study demonstrates how AI may be used in patient education in PAD. Both ChatGPT and Gemini chatbots are adaptable and can tailor their responses to an intended audience. Both have shown the ability to generate mostly comprehensive and accurate answers as compared to responses generated by the Cleveland Clinic. This demonstrates that ChatGPT and Gemini can be valuable tools that providers can suggest to patients to augment their education, with appropriate provider oversight and an understanding of the limitations and risks of AI. The responses generated by both chatbots are above the national recommended grade reading level, which warrants improvement in AI-driven health communication. Given the growing reliance on AI in healthcare, further research should explore ways to enhance AI-generated medical content for broader patient accessibility and evaluate its impact on patient outcomes.
